# Lipocalin 2 promotes inflammatory breast cancer tumorigenesis and skin invasion

**DOI:** 10.1002/1878-0261.13074

**Published:** 2021-08-27

**Authors:** Emilly S. Villodre, Xiaoding Hu, Richard Larson, Pascal Finetti, Kristen Gomez, Wintana Balema, Shane R. Stecklein, Ginette Santiago‐Sanchez, Savitri Krishnamurthy, Juhee Song, Xiaoping Su, Naoto T. Ueno, Debu Tripathy, Steven Van Laere, François Bertucci, Pablo Vivas‐Mejía, Wendy A. Woodward, Bisrat G. Debeb

**Affiliations:** ^1^ Department of Breast Medical Oncology The University of Texas MD Anderson Cancer Center Houston TX USA; ^2^ MD Anderson Morgan Welch Inflammatory Breast Cancer Clinic and Research Program The University of Texas MD Anderson Cancer Center Houston TX USA; ^3^ Department of Radiation Oncology The University of Texas MD Anderson Cancer Center Houston TX USA; ^4^ Laboratory of Predictive Oncology Aix‐Marseille University Inserm CNRS Institut Paoli‐Calmettes CRCM Marseille France; ^5^ Department of Biological Sciences The University of Texas at Brownsville TX USA; ^6^ Department Biochemistry and Cancer Center University of Puerto Rico Medical Sciences Campus San Juan, Puerto Rico; ^7^ Department of Pathology The University of Texas MD Anderson Cancer Center Houston TX USA; ^8^ Department of Biostatistics The University of Texas MD Anderson Cancer Center Houston TX USA; ^9^ Department of Bioinformatics and Computational Biology The University of Texas MD Anderson Cancer Center Houston TX USA; ^10^ Center for Oncological Research (CORE) Integrated Personalized and Precision Oncology Network (IPPON) University of Antwerp Belgium

**Keywords:** brain metastasis, inflammatory breast cancer, LCN2, lipocalin 2, skin invasion

## Abstract

Inflammatory breast cancer (IBC) is an aggressive form of primary breast cancer characterized by rapid onset and high risk of metastasis and poor clinical outcomes. The biological basis for the aggressiveness of IBC is still not well understood and no IBC‐specific targeted therapies exist. In this study, we report that lipocalin 2 (LCN2), a small secreted glycoprotein belonging to the lipocalin superfamily, is expressed at significantly higher levels in IBC vs non‐IBC tumors, independently of molecular subtype. LCN2 levels were also significantly higher in IBC cell lines and in their culture media than in non‐IBC cell lines. High expression was associated with poor‐prognosis features and shorter overall survival in IBC patients. Depletion of LCN2 in IBC cell lines reduced colony formation, migration, and cancer stem cell populations *in vitro* and inhibited tumor growth, skin invasion, and brain metastasis in mouse models of IBC. Analysis of our proteomics data showed reduced expression of proteins involved in cell cycle and DNA repair in LCN2‐silenced IBC cells. Our findings support that LCN2 promotes IBC tumor aggressiveness and offer a new potential therapeutic target for IBC.

## Graphical abstract

In this study, the authors demonstrate that lipocalin 2 (LCN2) is highly upregulated in inflammatory breast cancer (IBC), a rare, rapidly‐progressing and aggressive variant of primary breast cancer. Depletion of LCN2 in IBC cell lines reduced colony formation, migration, and cancer stem cell populations *in vitro* and inhibited tumor growth, skin invasion, and brain metastasis in mouse models of IBC.

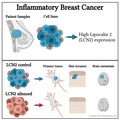

**Abbreviations**
BrMS: brain metastasis subline
ER: estrogen receptor
ERBB2/HER2: Erb‐B2 receptor tyrosine kinase 2
IBC: inflammatory breast cancer
LCN2: Lipocalin 2
LuMS: lung metastasis subline
MAPK: mitogen‐activated protein kinase
MMP‐9: matrix metallopeptidase 9
MTOR: mammalian target of rapamycin
non‐IBC: non‐inflammatory breast cancer
PR: progesterone receptor
RPS6KB1: ribosomal protein S6 kinase B1
shCtl: short hairpin RNA control
shLCN2: short hairpin RNA for Lipocalin 2
TNBC: triple‐negative breast cancer

## 1 Introduction

Inflammatory breast cancer (IBC) is the most aggressive and deadly variant of primary breast cancer. Although IBC is considered rare in the United States (1–4% of all breast cancer cases), it accounts for a disproportionate 10% of breast cancer‐related deaths because of its aggressive proliferation and metastasis and limited therapeutic options [1, 2, 3, 4, 5]. IBC disproportionately affects young and African American women [1, 6]. IBC is associated with unique clinical and biological features and a distinctive pattern of recurrence with high incidence in central nervous system, lung, and liver as first site of relapse [4, 7, 8]. Even with multimodality treatment strategies, survival rates for women with IBC are far lower than for those with other types of breast carcinoma (non‐IBC), with estimated 5‐year overall survival rates limited to 40% vs 63% for non‐IBC [4, 6, 7, 8, 9]. These features underscore the critical need to better define the mechanisms that drive the aggressive behavior of IBC and to develop novel agents to improve the overall prognosis for women with IBC. Efforts have been undertaken to identify pathways and therapeutic targets distinct to IBC and to better elucidate the mechanisms of IBC aggressiveness [10, 11, 12, 13, 14, 15]. However, the molecular and cellular basis for IBC aggressiveness remains unclear. Identification of specific targets and unraveling the mechanisms of growth and metastasis of this aggressive disease could lead to improvements in IBC patient survival.

Lipocalin 2 [LCN2, also known as neutrophil gelatinase‐associated Lipocalin (NGAL), siderocalin, or 24p3] is a 25‐kDa secreted glycoprotein that belongs to the lipocalin superfamily. LCN2 is known to sequester iron, as it binds siderophore‐complexed ferric iron with high affinity, and has significant roles in immune and inflammatory responses, angiogenesis, cell proliferation, survival, and resistance to anticancer therapies [16, 17, 18, 19, 20, 21]. LCN2 has been implicated in the progression of several types of human tumors, including breast cancer, through several mechanisms, such as stabilization of matrix metallopeptidase 9 (MMP‐9), sequestration of iron, induction of epithelial–mesenchymal transition, apoptosis resistance, lymphangiogenesis, and cell cycle arrest [16, 17, 18, 19, 20, 22, 23, 24, 25, 26]. Moreover, high LCN2 expression levels have been linked with poorer survival in patients with breast cancer [16, 26, 27, 28]. Little is known regarding the oncogenic role of LCN2 in IBC tumors.

In the present study, we demonstrate that LCN2 was expressed at significantly higher levels in patients with IBC and that LCN2 promoted tumor growth, skin invasion, and metastasis in xenograft mouse models of IBC.

## 2 Materials and methods

### 2.1 Cell lines

The SUM149 cell line was purchased from Asterand (Detroit, MI, USA), and MDA‐IBC3 cell line was generated in Dr. Woodward's laboratory [29, 30] and cultured in Ham's F‐12 media supplemented with 10% FBS (GIBCO, Thermo Fisher, Carlsbad, CA, USA), 1 µg·mL^−1^ hydrocortisone (#H0888, Sigma‐Aldrich, St. Louis, MO, USA), 5 µg·mL^−1^ insulin (#12585014; Thermo Fisher), and 1% antibiotic‐antimycotic (#15240062; Thermo Fisher). HEK293T cells were purchased from the American Type Culture Collection (Manassas, VA, USA) and cultured in Dulbecco's modified Eagle's medium supplemented with 10% FBS and 1% penicillin and streptomycin (#15140122; Invitrogen, Carlsbad, CA, USA). All cell lines were kept at 37 °C in a humidified incubator with 5% CO_2_ and were authenticated by short tandem repeat profiling at the Cytogenetics and Cell Authentication Core at UT MD Anderson Cancer Center.

### 2.2 Lentivirus‐mediated knockdown

LCN2 stable knockdown clones were generated in SUM149 or MDA‐IBC3 cells by using shRNA (shLCN2‐1: TRCN0000060289 from Sigma‐Aldrich; shLCN2‐2: RHS4430‐200252675 or shLCN2‐3: RHS4430‐200246537 from MD Anderson's Functional Genomics Core Facility, Houston, TX, USA). The MISSION(R) pLKO.1‐puro Empty Vector (SHC001, Sigma) was used as control (shCtl). HEK293T cells were transfected with 4.05 µg of target plasmid, pCMV‐VSV‐G (0.45 µg; #8584; Addgene, Watertown, MA, USA) and pCMV delta R8.2 (3.5 µg, #12263, Addgene) by using Lipofectamine 2000 (Life Technologies, Carlsbad, CA, USA) for 24 h. SUM149 and MDA‐IBC3 cells were incubated with the supernatant‐containing virus plus 8 µg·mL^−1^ of polybrene for 24 h. Stable cell lines were selected with 1 μg·mL^−1^ of puromycin.

### 2.3 RNA isolation and real‐time PCR

RNA was isolated by using TRIzol Reagent (Life Technologies) according to the manufacturer's instructions. The cDNA was obtained with a High Capacity cDNA Reverse Transcription Kit with RNase Inhibitor (Thermo Fisher Scientific). Real‐time PCR was done by using Power SYBR Green PCR Master Mix (Applied Biosystems, Foster City, CA, USA) on a 7500 Real‐Time PCR system (Applied Biosystems). LCN2 forward primer: 3′‐CCACCTCAGACCTGATCCCA‐5′, reverse primer: 3′‐ CCCCTGGAATTGGTTGTCCTG‐5′; GAPDH forward primer: 3′‐GAAGGTGAAGGTCGGAGT‐5′, reverse primer: 3′‐GAAGATGGTGATGGGATTTC‐5′.

### 2.4 ELISA

Human LCN2/NGAL Quantikine ELISA Kits (#DLCN20; R&D Systems, Minneapolis, MN, USA) were used to measure the levels of LCN2 in the cell lines according to the manufacturer's instructions. Samples were assayed in duplicate.

### 2.5 Western blotting

Cells were lysed in RIPA buffer (Sigma) supplemented with 10 µL·mL^−1^ phosphatase and 10 µL·mL^−1^ protease inhibitor cocktail. SDS/PAGE and immunoblotting were carried out as described elsewhere [29]. The following primary antibodies were used: LCN2 antibody (1 : 1000, #MAB1757SP; R&D Systems), pMEK1/2 (1 : 1000, #9154; Cell Signaling, Danvers, MA, USA), MEK1/2 (1 : 1000, # 8727; Cell Signaling), pERK1/2 (1 : 1000, # 4370; Cell Signaling), ERK1/2 (1 : 1000, # 9102; Cell Signaling), or GAPDH (1 : 5000, #5174; Cell Signaling), and samples were incubated overnight at 4 °C. Secondary antibodies (1 : 5000), anti‐rat IgG (#HAF005; R&D Systems) and anti‐rabbit IgG (#7074; Cell Signaling) were incubated with the samples for 2 h at room temperature. Immunoreactivity was visualized with Clarity™ Western ECL Substrate (#1705061; Bio‐Rad, Hercules, CA, USA) using ImageQuant LAS4000 (GE Healthcare, Chicago, IL, USA).

### 2.6 Proliferation

About 2500 cells were seeded in triplicate in a 96‐well plate. Cell proliferation was measured every day for up to 72 h with the CellTiter‐Blue assay (#G8080; Promega, Madison, WI, USA) according to the manufacturer's instructions. Absorbance was recorded at OD595 nm with a Multifunctional Reader VICTOR X 3 (PerkinElmer, Waltham, MA, USA).

### 2.7 Colony‐formation assay

About 100 SUM149 or 500 MDA‐IBC3 shRNA Control or LCN2‐silenced cells were plated in triplicate in 6‐well plates. After 15 days, cells were fixed with methanol for 2 min and stained with 0.2% (w/v) crystal violet for 30 min. Colonies were counted using GelCount (Oxford Optoronix, Abingdon, UK).

### 2.8 Migration and invasion assay

For the migration assay, 50 000 cells per well (triplicate) were seeded in medium without serum onto 8 μm polypropylene filter inserts in Boyden chambers (Fisher). Medium with 10% FBS was added onto the well. After 24 h, cells on the bottom of the filter were fixed and stained with Thermo Scientific Shandon Kwik Diff Stains (Fisher). The invasion assay was done as described above, except that the 8 μm polypropylene filter inserts were coated with Matrigel (#CB‐40234; Corning Inc., Corning, NY, USA) and incubated for 24 h. Ten visual fields were randomly chosen under microscopy and cells were quantified by using imagej software (National Institutes of Health, Bethesda, MD, USA).

### 2.9 Mammosphere assay

For primary mammosphere formation, 30 000 SUM149 or MDA‐IBC3 control or LCN2‐knockdown cells were plated in ULTRALOW attachment six‐well plates (Corning Inc.) in mammosphere medium [serum‐free MEM supplemented with 20 ng·mL^−1^ of bFGF (Gibco), 20 ng·mL^−1^ epidermal growth factor (Gibco), B27 1× (Gibco), and gentamycin/penicillin/streptomycin (Thermo Fisher)]. After 7 days, 5 μg·mL^−1^ of MTT (Sigma‐Aldrich) was added for 30 min and the mammospheres were counted using GelCount (Oxford Optoronix). For secondary mammosphere formation, primary mammospheres were dissociated and counted, and 10 000 cells were plated in the ULTRALOW attachment six‐well plates in mammosphere media and analyzed after 7 days.

### 2.10 CD44/CD24 flow cytometry

About 2.5 × 10^5^ cells were suspended in CD24‐PE mouse anti‐human (#555428; BD Biosciences) or CD24‐BV421 Mouse Anti‐Human (#562789; BD Biosciences, Franklin Lakes, NJ, USA) and CD44‐FITC mouse anti‐human (#555478; BD Biosciences) or CD44‐APC Mouse anti‐Human (#559942; BD Biosciences) solutions and incubated for 20 min on ice. Cells only, PE/BV421 only, and FITC/APC only were used as controls to set the gating. Fluorescence was detected by using a Gallios Flow Cytometer (Beckman Coulter, Brea, CA, USA) at the Flow Cytometry and Cellular Imaging Core Facility (UT MD Anderson Cancer Center, Houston, TX, USA). flowjo software (Treestar, Ashland, OR, USA) was used to analyze the data.

### 2.11 Kinase enrichment analysis

The RPPA data were also used for the phosphoproteomic analysis using kinase enrichment analysis (KEA—https://maayanlab.cloud/kea3/) [31]. Briefly, the 20 proteins that exhibit the highest phosphorylation fold change levels in control vs LCN2‐silenced cells were analyzed. Two different analyses were performed using KEA: (a) The differentially phosphorylated proteins are queried for enrichment of kinase substrates and (b) the differentially phosphorylated proteins are queried for enrichment of interacting proteins across seven databases. The latter analysis is more general and is not limited to only kinase substrates. Both analyses result in the detection of kinases that are putatively responsible for the observed phosphorylation differences. Identified proteins by both analyses were mapped onto the STRING network (https://string‐db.org) to investigate their mutual interactions.

### 2.12 *In vivo* experiments

Four‐ to six‐week‐old female athymic SCID/Beige mice were purchased from Harlan Laboratories (Indianapolis, IN, USA) and allowed to acclimate for 1 week before use. All mice were given free access to food and water in a specific pathogen‐free condition. All animal experiments were done in accordance with protocols approved by the Institutional Animal Care and Use Committee of MD Anderson Cancer Center. Mice were euthanized with overdose of isoflurane when they met the institutional criteria for tumor size and overall health condition. For primary tumor growth, cells were injected into the orthotopic cleared mammary fat pad of mice as previously described [32]. Briefly, 5 × 10^5^ SUM149 shRNA Control / LCN2‐knockdown cells were injected (9 mice/Control; 10 mice/LCN2 KD). Tumor volumes were assessed weekly by measuring palpable tumors with calipers. Volume (*V*) was determined as *V* = (*L* × *W* × *W*) × 0.5, with *L* being length and *W* width of the tumor. To determine latency, the first day when palpable tumors appeared was used to plot the graph. For brain metastatic colonization studies, we followed our laboratory protocol [33]. Briefly, 1 × 10^6^ MDA‐IBC3 GFP‐labeled shRNA Control/LCN2‐knockdown cells (10 mice/group) were injected via the tail vein into SCID/Beige mice. At 12 weeks after tail‐vein injection, mice were euthanized, and brain tissue collected and imaged with fluorescent stereomicroscopy (SMZ1500; Nikon Instruments, Melville, NY, USA). imagej was used to measure GFP‐positive areas to quantify the area of brain tumor burden. For mice with more than one brain metastasis, the area of each metastasis was considered and measured.

### 2.13 Statistical analysis

All *in vitro* experiments were repeated at least three times, and graphs depict mean ± SEM. Statistical significance was determined with Student's *t*‐tests (unpaired, two‐tailed) unless otherwise specified. One‐way analysis of variance was used for multiple comparisons. Mann–Whitney test was used when normality was not met. *LCN2* expression in breast cancer samples was analyzed in the IBC Consortium dataset [34] for IBC and from a meta‐dataset previously published [35]. Tumor samples were stratified as *LCN2*‐high when expression in tumor was at least two‐fold the mean expression level measured in the normal breast samples; otherwise, the sample was classified as *LCN2*‐low. Kaplan–Meier curves and log‐rank tests were used to compare survival distributions. Univariate and multivariate Cox regression models were used to evaluate the significance of LCN2 expression on overall survival. A *P* value of < 0.05 was considered significant. graphpad software (GraphPad Prism 8, La Jolla, CA, USA) was used.

## 3 Results

### 3.1 LCN2 mRNA is highly expressed in inflammatory breast cancer

**Fig. 1:**
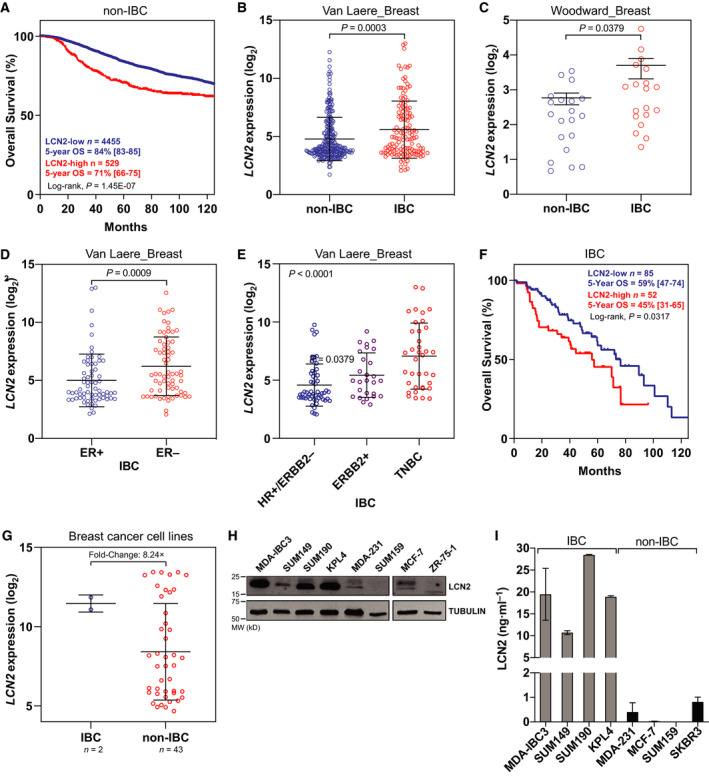
LCN2 was highly expressed in tumors from patients with IBC. (A) High *LCN2* expression was associated with shorter overall survival in a meta‐dataset of patients with non‐IBC. (B, C) *LCN2* mRNA expression was higher in tumors from IBC patients vs non‐IBC patients in two independent breast cancer datasets [IBC World Consortium Dataset; GSE45582]. (D) *LCN2* mRNA expression was higher in ER‐negative compared to ER^+^ samples IBC samples. (E) *LCN2* mRNA expression was higher in more aggressive molecular subtypes, ERBB2^+^ and TNBC, compared to HR‐positive/HERBB2‐negative subtype. (F) *LCN2*‐high expression correlates with shorter overall survival in patients with IBC. (G) *LCN2* mRNA expression was higher in IBC cell lines compared to non‐IBC cell lines. (H, I) LCN2 protein expression was higher in IBC cell lines compared to non‐IBC cell lines shown by (H) immunoblotting or (I) ELISA for secreted LCN2 in supernatants. Bar graphs indicate mean ± SEM from three independent experiments. graphpad prism software was used to obtain the *P* values, with Mann–Whitney tests used to compare two categories or one‐way analysis of variance to compare three or more categories. Black lines in each group (B–E, and G) indicate mean ± SD.

Previous studies have shown that high LCN2 expression levels were correlated with poor prognosis in breast cancer patients [17, 25, 26, 27]. We further validated these findings by analyzing a meta‐dataset of 8951 breast cancers, in which 87% of tumor samples were classified as *LCN2*‐low (*n* = 7830/8951) and 13% as *LCN2*‐high (*n* = 1121/8951). Table 1 summarizes the clinico‐pathological patient characteristics stratified by *LCN2* expression status. High expression of *LCN2* was associated with variables commonly associated with poor outcome: younger patients' age, high grade, advanced stage tumors (pN‐positive and pT3), ductal type, estrogen receptor (ER)‐negative status, progesterone receptor (PR)‐negative status, Erb‐B2 receptor tyrosine kinase 2 (ERBB2)‐positive status, and aggressive molecular subtypes [ERBB2^+^ and triple‐negative breast cancer (TNBC) subtypes]. In this cohort, we also analyzed the association of *LCN2* expression and survival over time using the Kaplan–Meier method. We found that *LCN2*‐high tumors had significantly shorter overall survival (*P* < 0.0001) than *LCN2*‐low tumors (Fig. 1A).

**Table 1:** Clinico‐pathological characteristics of tumor samples from patients with IBC or non‐IBC according to *LCN2* expression. The percentage between brackets is relative to the total number of samples informative in each column.

Characteristics	Level	All (*n* = 8951)	*LCN2*‐low (*n* = 7830)	*LCN2*‐high (*n* = 1121)	*P* value
Age (years)	≤ 50	2587 (36%)	2218 (36%)	369 (42%)	1.10E‐04
	> 50	4520 (64%)	4018 (64%)	502 (58%)	
Pathological grade	1	717 (11%)	680 (13%)	37 (4%)	< 1.00E‐06
	2	2549 (41%)	2359 (43%)	190 (22%)	
	3	3016 (48%)	2389 (44%)	627 (73%)	
Pathological node (pN)	Negative	3666 (57%)	3253 (57%)	413 (53%)	3.89E‐02
	Positive	2788 (43%)	2426 (43%)	362 (47%)	
Pathological size (pT)	pT1	2116 (37%)	1912 (38%)	204 (31%)	2.00E‐06
	pT2	2931 (52%)	2588 (52%)	343 (53%)	
	pT3	604 (11%)	498 (10%)	106 (16%)	
Pathological type	Ductal	4027 (79%)	3492 (78%)	535 (86%)	3.00E‐06
	Lobular	500 (10%)	471 (11%)	29 (5%)	
	Other	574 (64%)	519 (12%)	55 (9%)	
ER status^a^	Negative	2753 (31%)	1955 (25%)	798 (71%)	1.97E‐215
	Positive	6198 (69%)	5875 (75%)	323 (29%)	
PR status^a^	Negative	4635 (52%)	3746 (48%)	889 (80%)	3.06E‐86
	Positive	4284 (48%)	4055 (52%)	229 (20%)	
ERBB2 status^a^	Negative	7862 (88%)	6975 (89%)	887 (79%)	2.37E‐21
	Positive	1089 (12%)	855 (11%)	234 (21%)	
HR subtype^a^	HR^+^/ERBB2^−^	5914 (66%)	5598 (72%)	316 (28%)	< 1.00E‐06
	ERBB2^+^	1089 (12%)	855 (11%)	234 (21%)	
	TNBC	1938 (22%)	1368 (17%)	570 (51%)	
Overall survival^b^		4984	1.00	1.58 (1.34–1.86)^c^	3.31E‐08

^a^ mRNA status.

^b^ Univariate analysis.

^c^ Hazard ratio (95% confidence interval)

Analysis of microarray data from the IBC World Consortium Dataset [34] consisting of IBC and non‐IBC patient samples (*n* = 389; IBC = 137, non‐IBC = 252) showed that *LCN2* expression was significantly higher in tumors from IBC patients compared to non‐IBC (*P* = 0.0003; Fig. 1B). We validated this finding in another independent dataset [36] that compared mRNA expression of microdissected IBC and non‐IBC tumors (*P* = 0.0379; Fig. 1C). Here too, *LCN2* expression was higher in ER‐negative IBC patients compared to ER‐positive (*P* = 0.0009; Fig. 1D) and in more aggressive subtypes, ERBB2‐positive and TNBC, compared to hormone receptor (HR)‐positive/ERBB2‐negative subtype (Fig. 1E). Multivariate analysis showed that *LCN2* was expressed significantly higher in IBC tumors relative to non‐IBC tumors, independently from the molecular subtype differences (Odds ratio, 1.71, *P* = 0.034; Table 2). Here too, the survival analysis in IBC patients showed that *LCN2*‐high tumors had significantly shorter overall survival (*P* = 0.0317) than *LCN2*‐low tumors (Fig. 1F). Consistent with the patient data, the levels of *LCN2* were higher in IBC cell lines (Fig. 1G) and in the supernatants collected from IBC cell lines relative to non‐IBC (Fig. 1H). Taken together, our findings show that LCN2 is highly expressed in IBC tumors and is correlated with aggressive features and poor outcome suggesting it may contribute to the aggressive pathobiology of IBC tumors.

**Table 2:** Univariate and multivariate Cox regression analysis of IBC patient samples vs non‐IBC (*n* = 389).

IBC vs non‐IBC	Univariate			Multivariate		
	Odds ratio	95% CI	*P* value	Odds ratio	95% CI	*P* value
LCN2, high vs low	2.09	1.43–3.06	1.43E‐03	1.71	1.13–2.6	3.42E‐02
Molecular subtype						
ERBB2^+^ vs HR^+^/ERBB2^−^	2.82	1.82–4.38	1.02E‐04	2.5	1.59–3.93	8.16E‐04
TNBC vs HR^+^/ERBB2^−^	1.9	1.22–2.97	1.69E‐02	1.51	0.93–2.44	0.162

### 3.2 LCN2‐knockdown reduced aggressiveness features *in vitro*

**Fig. 2:**
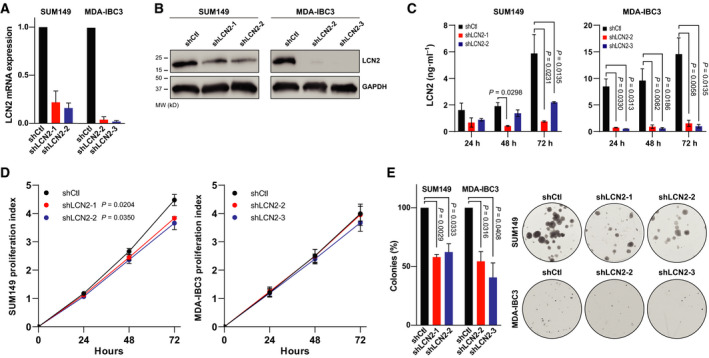
Silencing LCN2 decreased colony formation efficiency. LCN2 was knocked down (shLCN2) in two IBC cell lines (SUM149 and MDA‐IBC3) and confirmed by (A) qRT‐PCR and (B) immunoblotting. (C) Secreted LCN2 measured in control and silenced cells by ELISA at the indicated times. Bar graphs indicate mean ± SEM, calculated after three independent experiments; *P* values from *t*‐tests. (D) Proliferation was evaluated in control and LCN2‐silenced SUM149 and MDA‐IBC3 cells with CellTiter‐Blue assay on the indicated days. *P* values from *t*‐tests. (E) Cells were seeded in low numbers to measure the capacity to form colonies in LCN2 knockdown and control. Bar graphs indicate mean ± SEM, calculated after three independent experiments; *P* values from *t*‐tests.

**Fig. 3:**
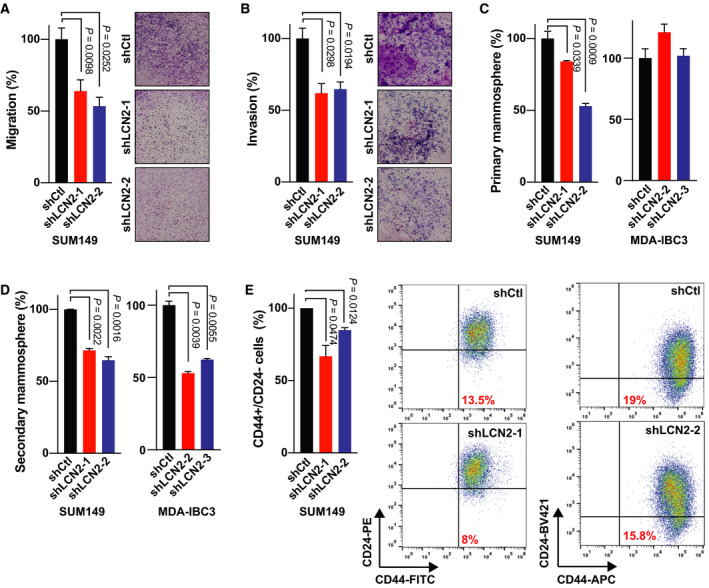
LCN2 knockdown reduced aggressiveness features *in vitro*. (A) Migration and (B) invasion by control cells (shCtl) and LCN2‐knockdown (shLCN2) SUM149 cells. (C) Primary mammosphere formation efficiency and (D) secondary mammosphere formation efficiency. (E) CD44^+^CD24^−^ cells (marker of cancer stem cells) were measured by flow cytometry. Bar graphs indicate mean ± SEM, calculated after three independent experiments; *P* values from *t*‐tests.

We generated stable LCN2‐knockdown cell lines [SUM149 (triple‐negative IBC); MDA‐IBC3 (HER2^+^ IBC)] to investigate the role of LCN2 in IBC aggressiveness *in vitro* and *in vivo*. LCN2‐knockdown was confirmed by qRT‐PCR and immunoblotting (Fig. 2A,B). Because LCN2 is a secreted protein, we evaluated levels of LCN2 protein in the supernatants from control and LCN2‐silenced IBC cell lines by using ELISA. We observed significant reduction of secreted LCN2 in the LCN2‐silenced IBC cells (Fig. 2C). Silencing LCN2 slightly reduced proliferation of SUM149 cells but did not affect MDA‐IBC3 cells (Fig. 2D). Depletion of LCN2 reduced the capacity of the cells to form colonies (Fig. 2E) and to migrate and invade (Fig. 3A,B). LCN2 silencing also significantly reduced the percentage of cancer stem cell populations in LCN2‐silenced IBC cells relative to control, as shown by reductions in primary and secondary mammosphere formation efficiency (Fig. 3C,D) and CD44^+^CD24^−^ cell subpopulations (Fig. 3E). These findings indicate that suppression of LCN2 in IBC cells reduced *in vitro* aggressiveness features.

### 3.3 Silencing of LCN2 inhibited tumor growth and skin invasion

**Fig. 4:**
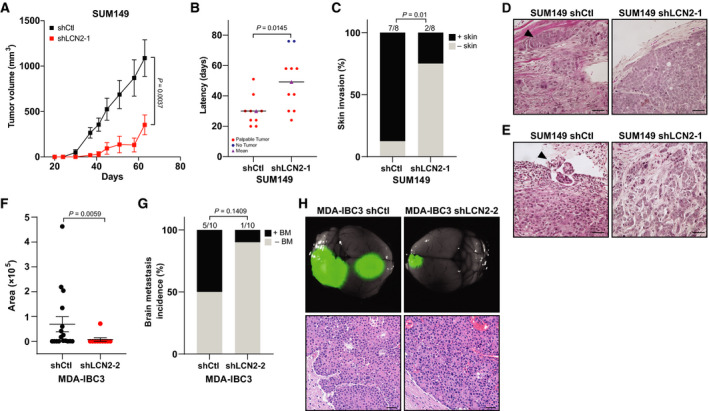
Silencing LCN2 inhibited tumor growth and skin invasion. (A–C) SUM149 shRNA Ctl or LCN2‐knockdown (shLCN2) cells were transplanted orthotopically into the cleared mammary fat pad of SCID/Beige mice (*n* = 9/shCtl; 10/shLCN2) and tumor volume measured weekly; (A) tumor volume, (B) tumor latency, and (C) incidence of skin invasion/recurrence after resection of primary tumors. (A, B) *P* values from *t*‐tests and (C) from Fisher's exact test. (D, E) Hematoxylin and eosin staining of primary tumors generated from LCN2‐control and knockdown SUM149 cells. Both (D) skin invasion and (E) tumor emboli, two hallmarks of IBC, appeared only in the control‐derived tumors (arrowhead). Scale bar, 100 µm. (F) Metastatic burden (area) of each brain metastasis formed was quantified by using imagej software. BM, brain metastasis. *P* values from *t*‐tests. (G) Incidence of brain metastasis. *N* = 10 mice per group. Fisher's exact test was used to obtain *P* values. (H) Top, GFP imaging of brain metastasis lesions generated from tail‐vein injection of GFP‐labeled MDA‐IBC3 shRNA Ctl or LCN2‐knockdown cells, and bottom, hematoxylin and eosin stains of brain metastasis lesions. Scale bar, 50 µm.

To investigate the effects of LCN2 on tumor growth and skin invasion, key characteristics of IBC tumors [4], we injected SUM149 control or LCN2‐silenced cells into the cleared mammary fat pad of SCID/Beige mice. Silencing of LCN2 reduced tumor volumes (*P* = 0.0037; Fig. 4A) and tumor latency, that is, the ability to initiate tumor growth: mice transplanted with SUM149 LCN2‐silenced cells took longer to initiate tumors than did those transplanted with SUM149 control cells (*P* = 0.0145; Fig. 4B). Because IBC typically manifests with skin invasion and formation of tumor emboli [4], we assessed skin invasion visually during primary tumor growth, as evidenced by loss of fur at the tumor site and skin redness and thickness, and during tumor excision when tumors were firmly connected with the skin. Analysis of resected tumors showed that significantly fewer mice with SUM149 LCN2‐silenced cells had skin invasion/recurrence compared with mice implanted with control cells [shLCN2: two of eight mice (25%) vs shControl: seven of eight mice (87.5%), *P* = 0.01; Fig. 4C,D]. On histologic examination, tumors generated from LCN2‐silenced cells were more differentiated than those generated from control SUM149 cells (Fig. 4E); we further observed tumor emboli, another hallmark of IBC tumors, in SUM149 control‐transplanted tumors but not in tumors generated from LCN2‐silenced SUM149 cells (Fig. 4E).

We recently generated xenograft mouse models of brain and lung metastasis via tail‐vein injection of IBC cell lines [29, 33]. We also showed that sublines of SUM149 generated from brain metastases (BrMS) and lung metastases (LuMS) have distinct morphologic and molecular features [29]. Microarray profiling of these sublines showed upregulation of *LCN2* in the brain metastatic sublines (Fig. S1A), and we confirmed higher levels of secreted LCN2 in the BrMS sublines vs LuMS by ELISA (Fig. S1B). Most recently, Chi *et al*. [37] elegantly demonstrated that LCN2 promotes brain metastatic growth in mouse models of leptomeningeal metastasis, highlighting a potential brain metastasis‐promoting role for LCN2. We investigated the functional role of LCN2 in IBC brain metastasis by using our HER2^+^ MDA‐IBC3 mouse model, which has a high propensity to metastasize to the brain and has been used to identify targets and develop therapeutics against brain metastasis [29, 38, 39, 40]. We found that the brain metastatic burden was significantly lower in mice that had received tail‐vein injection of LCN2‐silenced MDA‐IBC3 cells than in mice injected with control cells (Fig. 4F, *P* = 0.0059). Also, fewer mice injected with LCN2‐silenced cells developed brain metastasis [one of 10 (10%)] than did mice injected with control cells [five of 10 mice (50%)], although this trend was not statistically significant (*P* = 0.1409; Fig. 4G). Representative stereofluorescence and hematoxylin and eosin images of brain metastasis are shown in Fig. 4H. Overall, our findings suggest that LCN2 may drive IBC tumor progression, skin invasion/recurrence, and brain metastasis.

### 3.4 LCN2 silencing impairs cell cycle‐associated proteins

**Fig. 5:**
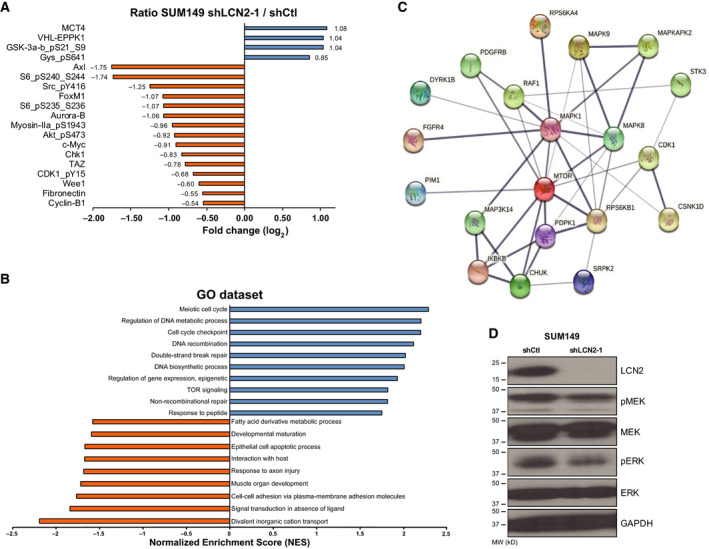
Silencing of LCN2 impairs cell cycle‐associated proteins. (A) The top proteins downregulated in LCN2‐silenced cells compared with control cells after RPPA proteomic analysis. (B) Gene set enrichment analysis of RPPA data identified pathways that are enriched or downregulated in control vs LCN2‐silenced SUM149 cells. (C) STRING interaction network of predicted active kinases based on enrichment of kinase substrates and protein interactions identified using KEA. The confidence of the interaction is reflected by the edge thickness. Based on node distribution analysis, four central proteins were identified (MAPK1, MAPK8, RPS6KB1, and MTOR). (D) Silencing of LCN2 in SUM149 cells reduced pMEK and pERK expression.

To identify potential mechanisms and pathways involved in suppression of tumor growth and skin invasion in LCN2‐silenced cells, we used reverse‐phase proteomics assay (RPPA) profiling to compare control and LCN2‐silenced SUM149 cells. Our analysis showed reduced expression of cell cycle‐associated proteins [such as AXL, FOXM1, Chk1, CDK1, Wee1, Aurora‐B, and cyclin‐B1 and the mammalian target of rapamycin (mTOR)/AKT pathway] in LCN2‐silenced IBC cells (Fig. 5A). Gene set enrichment analysis revealed several key signaling pathways that were enriched in the control cells, including those associated with cell cycling, DNA repair, and mTOR signaling (Fig. 5B). Furthermore, we performed KEA [31] on the 20 proteins that exhibited the highest phosphorylation fold changes in LCN2‐control vs LCN2‐silenced SUM149 cells (Table S1). Based on the set of predicted activated kinases (Tables S1 and S2), an interaction network was generated (Fig. 5C). Based on the node degree distribution (i.e., the distribution of the number of interactions per gene in the network), mitogen‐activated protein kinase 1 (MAPK1; *N* = 10), MAPK8 (*N* = 7), ribosomal protein S6 kinase B1 (RPS6KB1; *N* = 7), and MTOR (*N* = 11) appear to be central to LCN2 action in SUM149 cells. Our immunoblotting experiments confirmed that silencing LCN2 reduces the phosphorylated forms of MEK (pMEK) and ERK (pERK) of the MAPK signaling pathway (Fig. 5D). Thus, LCN2 may regulate different pathways, including cell cycle, MAPK, and mTOR proteins to promote tumor growth in IBC.

## 4 Discussion

Inflammatory breast cancer is an aggressive form of breast cancer with poor survival outcomes. Although considerable effort has been undertaken to understand the unique biology of IBC, insights are still limited as to the molecular properties that mediate the development and aggressiveness of IBC. Herein, we report that the secreted glycoprotein LCN2 was highly expressed in tumors from IBC patients and in IBC cell lines. We further demonstrate, with *in vitro* and *in vivo* studies, that LCN2 has a tumor promoter function in IBC.

LCN2 has been implicated in the progression of several types of human tumors. LCN2 expression is higher in solid tumors than in corresponding normal tissues [25, 41], and it is mainly described as tumor promoter in many cancers, including pancreas, glioblastoma, thyroid, kidney, esophagus, and breast cancer [19, 28, 42, 43, 44, 45, 46, 47, 48].

In breast cancer, increased LCN2 expression was associated with poor outcomes and shown to be an independent prognostic marker of disease‐specific‐free survival [27, 48, 49]. LCN2 also correlates with several important unfavorable prognostic factors in breast cancer, such as hormone‐negative status, high proliferation levels, high histologic grade, and the presence of lymph node metastases [27, 48, 49]. Further, serum levels of LCN2 have been shown to correlate with cancer progression and higher likelihood of metastasis in breast cancer [26, 50]. The oncogenic role of LCN2 has been reported in xenograft and LCN2‐knockout mouse models. Disruption of the *LCN2* gene in MMTV‐PyMT mice was found to suppress primary tumor formation without affecting lung metastasis [51]. Using the spontaneous MMTV‐*ErbB2*(V664E) LCN2^−/−^ mouse model, Leng *et al*. [18] reported delayed tumor growth and reduced lung metastasis burden in these LCN2^−/−^ mice. Another group showed that injection of wild‐type PyMT tumor cells into LCN2‐deficient mice did not alter primary tumor formation but did significantly reduce lung metastasis [52]. LCN2 has also been shown to promote tumor progression in xenograft mouse models [16, 26]. Consistent with these studies, our current work with xenograft mouse models of IBC supports that LCN2 has a tumor promoter function in IBC tumors. We demonstrated that silencing of LCN2 reduced tumor initiation and growth, skin invasion/recurrence, and brain metastasis burden in preclinical mouse models of IBC.

We further reported that depletion of LCN2 in IBC cell cultures reduced features associated with aggressiveness *in vitro*, including migration, invasion, and cancer stem cell populations. Others have also found that reduction of LCN2 levels affected the same features in MDA‐MB‐231 cells (TNBC cell line) and in SK‐BR‐3 (HER2^+^ breast cancer cell line) [18, 26]. However, our data demonstrating higher levels of secreted LCN2 in IBC vs non‐IBC cell lines and showing significant inhibition of key IBC tumor features such as tumor emboli/skin invasion in LCN2‐silenced tumors suggest that LCN2 may exert its influence via an IBC‐specific mechanism. The LCN2 protein has many functions, including transport of fatty acids and iron, induction of apoptosis, suppression of bacterial growth, and modulation of inflammatory responses [16, 17, 18, 19, 20, 26, 53]. In malignant cells, LCN2 promotes oncogenesis through several mechanisms, including stabilization of MMP‐9, sequestration of iron, induction of EMT, apoptosis resistance, and regulation of cell cycling [16, 17, 18, 19, 20, 26, 53]. Here, we report that LCN2 could regulate cell cycle‐associated proteins such as FOXM1, Chk1, CDK1, Aurora‐B, Wee1, and cyclin‐B1 to promote its oncogenic role in IBC tumors. Others have also found that silencing of LCN2 affected the expression of cell cycle proteins by reducing cyclin‐D1 and inducing p21, resulting in G0‐G1 cell cycle arrest [22, 24, 25].

LCN2 is also a potential therapeutic target in cancer and other diseases. An antibody against LCN2 was found to decreased lung metastasis in a 4T1‐induced aggressive mammary tumor model [18]. In cervical cancer cells, treatment with LCN2‐neutralizing antibody reduced the migration and invasion of cells that overexpressed LCN2 [54]. In other diseases, use of an anti‐LCN2‐neutralizing antibody showed reductions in reperfusion injury after stroke and attenuated skin lesions in a psoriasis mouse model [55, 56]. These findings suggest that LCN2 could be an exploitable therapeutic target in IBC and other aggressive tumors. Further studies are needed to explore therapeutic strategies in IBC models by using antibodies against LCN2 or targeting LCN2‐associated molecular pathways, including those involved in cell cycling.

## 5 Conclusion

In summary, our studies provide evidence, for the first time, that LCN2 is highly upregulated in IBC tumors and that it is required for tumor growth and skin invasion in mouse models of IBC; our findings further suggest that LCN2 could be a therapeutic target for IBC and other aggressive cancers.

## Author contributions

ESV and BGD conceived and designed the project, performed most of the experiments, analyzed the data, and interpreted the results. XH, RL, WB, SRS, and KG performed some experiments. PF, FB, and XS helped with data analysis. JS provided statistical analysis support. SK provided pathological expertise and analysis of xenograft tumors. SVL, FB, GS‐S, PV‐M, NTU, WAW, and DT provided resources and contributed to revision of the manuscript. ESV and BGD wrote and edited the manuscript with input from all other authors.

## Conflict of interest

The authors declare no conflict of interest.

## Supporting information

**Fig. S1**. LCN2 expression is higher in sublines generated from brain metastasis (BrMS) than those generated from lung metastasis (LuMS). (A) Microarray analysis of sublines generated from BrMS or LuMS of SUM149 cells showed *LCN2* to be one of the top upregulated genes in BrMS (red arrow). Samples are described in Debeb 2016 [29]. (B) LCN2 is secreted in higher levels in BrMS vs LuMS.

**Table S1**. Top kinases predicted to be activated based on kinase‐substrate interactions of differentially phosphorylated proteins.

**Table S2**. Top kinases predicted to be activated based on kinase‐substrate and protein‐protein interaction analysis of differentially phosphorylated proteins across 10 different knowledge bases.

## Data Availability

The data that support the findings of this study are available from the corresponding author (bgdebeb@mdanderson.org) upon reasonable request. Additional data are available as supplementary material.

## References

[1] Chang S, Parker SL, Pham T, Buzdar AU & Hursting SD (1998) Inflammatory breast carcinoma incidence and survival: the surveillance, epidemiology, and end results program of the National Cancer Institute, 1975–1992. Cancer 82, 2366–2372. 9635529

[2] Dirix LY, Van Dam P, Prove A & Vermeulen PB (2006) Inflammatory breast cancer: current understanding. Curr Opin Oncol 18, 563–571. 16988576 10.1097/01.cco.0000245307.29026.0a

[3] Hance KW, Anderson WF, Devesa SS, Young HA & Levine PH (2005) Trends in inflammatory breast carcinoma incidence and survival: the surveillance, epidemiology, and end results program at the National Cancer Institute. J Natl Cancer Inst 97, 966–975. 15998949 10.1093/jnci/dji172 PMC2844937

[4] Robertson FM, Bondy M, Yang W, Yamauchi H, Wiggins S, Kamrudin S, Krishnamurthy S, Le‐Petross H, Bidaut L, Player AN*et al*. (2010) Inflammatory breast cancer: the disease, the biology, the treatment. CA Cancer J Clin 60, 351–375. 20959401 10.3322/caac.20082

[5] Wang Z, Chen M, Pan J, Wang X, Chen XS & Shen KW (2020) Pattern of distant metastases in inflammatory breast cancer ‐ a large‐cohort retrospective study. J Cancer 11, 292–300. 31897225 10.7150/jca.34572 PMC6930435

[6] Abraham HG, Xia Y, Mukherjee B & Merajver SD (2021) Incidence and survival of inflammatory breast cancer between 1973 and 2015 in the SEER database. Breast Cancer Res Treat 185, 229–238. 33033965 10.1007/s10549-020-05938-2

[7] Cristofanilli M, Valero V, Buzdar AU, Kau SW, Broglio KR, Gonzalez‐Angulo AM, Sneige N, Islam R, Ueno NT, Buchholz TA*et al*. (2007) Inflammatory breast cancer (IBC) and patterns of recurrence: understanding the biology of a unique disease. Cancer 110, 1436–1444. 17694554 10.1002/cncr.22927

[8] Fouad TM, Kogawa T, Liu DD, Shen Y, Masuda H, El‐Zein R, Woodward WA, Chavez‐MacGregor M, Alvarez RH, Arun B*et al*. (2015) Overall survival differences between patients with inflammatory and noninflammatory breast cancer presenting with distant metastasis at diagnosis. Breast Cancer Res Treat 152, 407–416. 26017070 10.1007/s10549-015-3436-x PMC4492876

[9] Dawood S, Ueno NT, Valero V, Woodward WA, Buchholz TA, Hortobagyi GN, Gonzalez‐Angulo AM & Cristofanilli M (2011) Differences in survival among women with stage III inflammatory and noninflammatory locally advanced breast cancer appear early: a large population‐based study. Cancer 117, 1819–1826, doi: 10.1002/cncr.25682 21509759

[10] Costa R, Santa‐Maria CA, Rossi G, Carneiro BA, Chae YK, Gradishar WJ, Giles FJ & Cristofanilli M (2017) Developmental therapeutics for inflammatory breast cancer: biology and translational directions. Oncotarget 8, 12417–12432. 27926493 10.18632/oncotarget.13778 PMC5355355

[11] Kleer CG, van Golen KL, Braun T & Merajver SD (2001) Persistent E‐cadherin expression in inflammatory breast cancer. Mod Pathol 14, 458–464. 11353057 10.1038/modpathol.3880334

[12] van Golen KL, Wu ZF, Qiao XT, Bao LW & Merajver SD (2000) RhoC GTPase, a novel transforming oncogene for human mammary epithelial cells that partially recapitulates the inflammatory breast cancer phenotype. Cancer Res 60, 5832–5838. 11059780

[13] Villodre ES, Gong Y, Hu X, Huo L, Yoon EC, Ueno NT, Woodward WA, Tripathy D, Song J & Debeb BG (2020) NDRG1 expression is an independent prognostic factor in inflammatory breast cancer. Cancers (Basel) 12, 3711. 10.3390/cancers12123711 PMC7763268 33321961

[14] Wang X, Semba T, Phi LTH, Chainitikun S, Iwase T, Lim B & Ueno NT (2020) Targeting signaling pathways in inflammatory breast cancer. Cancers 12, 2479. 10.3390/cancers12092479 PMC7563157 32883032

[15] Zhang D, LaFortune TA, Krishnamurthy S, Esteva FJ, Cristofanilli M, Liu P, Lucci A, Singh B, Hung MC, Hortobagyi GN*et al*. (2009) Epidermal growth factor receptor tyrosine kinase inhibitor reverses mesenchymal to epithelial phenotype and inhibits metastasis in inflammatory breast cancer. Clin Cancer Res 15, 6639–6648. 19825949 10.1158/1078-0432.CCR-09-0951 PMC2783487

[16] Fernandez CA, Yan L, Louis G, Yang J, Kutok JL & Moses MA (2005) The matrix metalloproteinase‐9/neutrophil gelatinase‐associated lipocalin complex plays a role in breast tumor growth and is present in the urine of breast cancer patients. Clin Cancer Res 11, 5390–5395. 16061852 10.1158/1078-0432.CCR-04-2391

[17] Iannetti A, Pacifico F, Acquaviva R, Lavorgna A, Crescenzi E, Vascotto C, Tell G, Salzano AM, Scaloni A, Vuttariello E*et al*. (2008) The neutrophil gelatinase‐associated lipocalin (NGAL), a NF‐kappaB‐regulated gene, is a survival factor for thyroid neoplastic cells. Proc Natl Acad Sci USA 105, 14058–14063. 18768801 10.1073/pnas.0710846105 PMC2544578

[18] Leng X, Ding T, Lin H, Wang Y, Hu L, Hu J, Feig B, Zhang W, Pusztai L, Symmans WF*et al*. (2009) Inhibition of lipocalin 2 impairs breast tumorigenesis and metastasis. Cancer Res 69, 8579–8584. 19887608 10.1158/0008-5472.CAN-09-1934

[19] Leung L, Radulovich N, Zhu CQ, Organ S, Bandarchi B, Pintilie M, To C, Panchal D & Tsao MS (2012) Lipocalin2 promotes invasion, tumorigenicity and gemcitabine resistance in pancreatic ductal adenocarcinoma. PLoS One 7, e46677. 23056397 10.1371/journal.pone.0046677 PMC3464270

[20] Shiiba M, Saito K, Fushimi K, Ishigami T, Shinozuka K, Nakashima D, Kouzu Y, Koike H, Kasamatsu A, Sakamoto Y*et al*. (2013) Lipocalin‐2 is associated with radioresistance in oral cancer and lung cancer cells. Int J Oncol 42, 1197–1204. 23403985 10.3892/ijo.2013.1815

[21] Yang J & Moses MA (2009) Lipocalin 2: a multifaceted modulator of human cancer. Cell Cycle 8, 2347–2352, doi: 10.4161/cc.8.15.9224 19571677 PMC3381736

[22] Chiang KC, Yeh TS, Wu RC, Pang JS, Cheng CT, Wang SY, Juang HH & Yeh CN (2016) Lipocalin 2 (LCN2) is a promising target for cholangiocarcinoma treatment and bile LCN2 level is a potential cholangiocarcinoma diagnostic marker. Sci Rep 6, 36138. 27782193 10.1038/srep36138 PMC5080596

[23] Jung M, Oren B, Mora J, Mertens C, Dziumbla S, Popp R, Weigert A, Grossmann N, Fleming I & Brune B (2016) Lipocalin 2 from macrophages stimulated by tumor cell‐derived sphingosine 1‐phosphate promotes lymphangiogenesis and tumor metastasis. Sci Signal 9, ra64, doi: 10.1126/scisignal.aaf3241 27353364

[24] Tung MC, Hsieh SC, Yang SF, Cheng CW, Tsai RT, Wang SC, Huang MH & Hsieh YH (2013) Knockdown of lipocalin‐2 suppresses the growth and invasion of prostate cancer cells. Prostate 73, 1281–1290, doi: 10.1002/pros.22670 23775308

[25] Xu J, Lv S, Meng W & Zuo F (2020) LCN2 mediated by IL‐17 affects the proliferation, migration, invasion and cell cycle of gastric cancer cells by targeting SLPI. Cancer Manag Res 12, 12841–12849. 33364832 10.2147/CMAR.S278902 PMC7751782

[26] Yang J, Bielenberg DR, Rodig SJ, Doiron R, Clifton MC, Kung AL, Strong RK, Zurakowski D & Moses MA (2009) Lipocalin 2 promotes breast cancer progression. Proc Natl Acad Sci USA 106, 3913–3918. 19237579 10.1073/pnas.0810617106 PMC2656179

[27] Bauer M, Eickhoff JC, Gould MN, Mundhenke C, Maass N & Friedl A (2008) Neutrophil gelatinase‐associated lipocalin (NGAL) is a predictor of poor prognosis in human primary breast cancer. Breast Cancer Res Treat 108, 389–397. 17554627 10.1007/s10549-007-9619-3

[28] Candido S, Abrams SL, Steelman LS, Lertpiriyapong K, Fitzgerald TL, Martelli AM, Cocco L, Montalto G, Cervello M, Polesel J*et al*. (2016) Roles of NGAL and MMP‐9 in the tumor microenvironment and sensitivity to targeted therapy. Biochim Biophys Acta 1863, 438–448. 26278055 10.1016/j.bbamcr.2015.08.010

[29] Debeb BG, Lacerda L, Anfossi S, Diagaradjane P, Chu K, Bambhroliya A, Huo L, Wei C, Larson RA, Wolfe AR*et al*. (2016) miR‐141‐mediated regulation of brain metastasis from breast cancer. J Natl Cancer Inst 108, djw026. 10.1093/jnci/djw026 PMC5017951 27075851

[30] Klopp AH, Lacerda L, Gupta A, Debeb BG, Solley T, Li L, Spaeth E, Xu W, Zhang X, Lewis MT*et al*. (2010) Mesenchymal stem cells promote mammosphere formation and decrease E‐cadherin in normal and malignant breast cells. PLoS One 5, e12180. 20808935 10.1371/journal.pone.0012180 PMC2922340

[31] Lachmann A & Ma'ayan A (2009) KEA: kinase enrichment analysis. Bioinformatics 25, 684–686. 19176546 10.1093/bioinformatics/btp026 PMC2647829

[32] Debeb BG, Lacerda L, Xu W, Larson R, Solley T, Atkinson R, Sulman EP, Ueno NT, Krishnamurthy S, Reuben JM*et al*. (2012) Histone deacetylase inhibitors stimulate dedifferentiation of human breast cancer cells through WNT/beta‐catenin signaling. Stem Cells 30, 2366–2377, doi: 10.1002/stem.1219 22961641 PMC4545658

[33] Hu X, Villodre ES, Woodward WA & Debeb BG (2021) Modeling brain metastasis via tail‐vein injection of inflammatory breast cancer cells. J Vis Exp. 168, e62249. 10.3791/62249 PMC11824898 33616115

[34] Van Laere SJ, Ueno NT, Finetti P, Vermeulen P, Lucci A, Robertson FM, Marsan M, Iwamoto T, Krishnamurthy S, Masuda H*et al*. (2013) Uncovering the molecular secrets of inflammatory breast cancer biology: an integrated analysis of three distinct affymetrix gene expression datasets. Clin Cancer Res 19, 4685–4696. 23396049 10.1158/1078-0432.CCR-12-2549 PMC6156084

[35] Bertucci F, Finetti P, Goncalves A & Birnbaum D (2020) The therapeutic response of ER+/HER2‐ breast cancers differs according to the molecular Basal or Luminal subtype. NPJ Breast Cancer 6, 8. 32195331 10.1038/s41523-020-0151-5 PMC7060267

[36] Woodward WA, Krishnamurthy S, Yamauchi H, El‐Zein R, Ogura D, Kitadai E, Niwa S, Cristofanilli M, Vermeulen P, Dirix L*et al*. (2013) Genomic and expression analysis of microdissected inflammatory breast cancer. Breast Cancer Res Treat 138, 761–772. 23568481 10.1007/s10549-013-2501-6 PMC3677826

[37] Chi Y, Remsik J, Kiseliovas V, Derderian C, Sener U, Alghader M, Saadeh F, Nikishina K, Bale T, Iacobuzio‐Donahue C*et al*. (2020) Cancer cells deploy lipocalin‐2 to collect limiting iron in leptomeningeal metastasis. Science 369, 276–282. 32675368 10.1126/science.aaz2193 PMC7816199

[38] Fukumura K, Malgulwar PB, Fischer GM, Hu X, Mao X, Song X, Hernandez SD, Zhang XH, Zhang J, Parra ER*et al*. (2021) Multi‐omic molecular profiling reveals potentially targetable abnormalities shared across multiple histologies of brain metastasis. Acta Neuropathol 141, 303–321. 33394124 10.1007/s00401-020-02256-1 PMC7852029

[39] Smith DL, Debeb BG, Thames HD & Woodward WA (2016) Computational modeling of micrometastatic breast cancer radiation dose response. Int J Radiat Oncol Biol Phys 96, 179–187. 27511855 10.1016/j.ijrobp.2016.04.014 PMC5702256

[40] Villodre ES, Hu X, Larson R, Eckhardt BL, Gong Y, Huo L, Song J, Krishnamurthy S, Ibrahim NK, Ueno NT*et al*. (2020) Ndrg1‐egfr axis in inflammatory breast cancer tumorigenesis and brain metastasis [abstract]. In: Proceedings of the 2019 San Antonio Breast Cancer Symposium; 2019 Dec 10‐14; San Antonio, TX, Vol. 80. AACR Cancer Res, Philadelphia, PA.

[41] Candido S, Maestro R, Polesel J, Catania A, Maira F, Signorelli SS, McCubrey JA & Libra M (2014) Roles of neutrophil gelatinase‐associated lipocalin (NGAL) in human cancer. Oncotarget 5, 1576–1594. 24742531 10.18632/oncotarget.1738 PMC4039233

[42] Du Z, Wu B, Xia Q, Zhao Y, Lin L, Cai Z, Wang S, Li E, Xu L, Li Y*et al*. (2019) LCN2‐interacting proteins and their expression patterns in brain tumors. Brain Res 1720, 146304. 31233712 10.1016/j.brainres.2019.146304

[43] Du ZP, Wu BL, Xie YM, Zhang YL, Liao LD, Zhou F, Xie JJ, Zeng FM, Xu XE, Fang WK*et al*. (2015) Lipocalin 2 promotes the migration and invasion of esophageal squamous cell carcinoma cells through a novel positive feedback loop. Biochim Biophys Acta 1853, 2240–2250. 26190820 10.1016/j.bbamcr.2015.07.007

[44] Gomez‐Chou SB, Swidnicka‐Siergiejko AK, Badi N, Chavez‐Tomar M, Lesinski GB, Bekaii‐Saab T, Farren MR, Mace TA, Schmidt C, Liu Y*et al*. (2017) Lipocalin‐2 promotes pancreatic ductal adenocarcinoma by regulating inflammation in the tumor microenvironment. Cancer Res 77, 2647–2660. 28249896 10.1158/0008-5472.CAN-16-1986 PMC5441230

[45] Miki M, Oono T, Fujimori N, Takaoka T, Kawabe K, Miyasaka Y, Ohtsuka T, Saito D, Nakamura M, Ohkawa Y*et al*. (2019) CLEC3A, MMP7, and LCN2 as novel markers for predicting recurrence in resected G1 and G2 pancreatic neuroendocrine tumors. Cancer Med 8, 3748–3760. 31129920 10.1002/cam4.2232 PMC6639196

[46] Santiago‐Sanchez GS, Pita‐Grisanti V, Quinones‐Diaz B, Gumpper K, Cruz‐Monserrate Z & Vivas‐Mejia PE (2020) Biological functions and therapeutic potential of lipocalin 2 in cancer. Int J Mol Sci 21, 4365. 10.3390/ijms21124365 PMC7352275 32575507

[47] Viau A, El Karoui K, Laouari D, Burtin M, Nguyen C, Mori K, Pillebout E, Berger T, Mak TW, Knebelmann B*et al*. (2010) Lipocalin 2 is essential for chronic kidney disease progression in mice and humans. J Clin Invest 120, 4065–4076. 20921623 10.1172/JCI42004 PMC2964970

[48] Wenners AS, Mehta K, Loibl S, Park H, Mueller B, Arnold N, Hamann S, Weimer J, Ataseven B, Darb‐Esfahani S*et al*. (2012) Neutrophil gelatinase‐associated lipocalin (NGAL) predicts response to neoadjuvant chemotherapy and clinical outcome in primary human breast cancer. PLoS One 7, e45826. 23056218 10.1371/journal.pone.0045826 PMC3467272

[49] Stoesz SP, Friedl A, Haag JD, Lindstrom MJ, Clark GM & Gould MN (1998) Heterogeneous expression of the lipocalin NGAL in primary breast cancers. Int J Cancer 79, 565–572. 9842963 10.1002/(sici)1097-0215(19981218)79:6<565::aid-ijc3>3.0.co;2-f

[50] Provatopoulou X, Gounaris A, Kalogera E, Zagouri F, Flessas I, Goussetis E, Nonni A, Papassotiriou I & Zografos G (2009) Circulating levels of matrix metalloproteinase‐9 (MMP‐9), neutrophil gelatinase‐associated lipocalin (NGAL) and their complex MMP‐9/NGAL in breast cancer disease. BMC Cancer 9, 390. 19889214 10.1186/1471-2407-9-390 PMC2775750

[51] Berger T, Cheung CC, Elia AJ & Mak TW (2010) Disruption of the Lcn2 gene in mice suppresses primary mammary tumor formation but does not decrease lung metastasis. Proc Natl Acad Sci USA 107, 2995–3000. 20133630 10.1073/pnas.1000101107 PMC2840296

[52] Oren B, Urosevic J, Mertens C, Mora J, Guiu M, Gomis RR, Weigert A, Schmid T, Grein S, Brune B*et al*. (2016) Tumour stroma‐derived lipocalin‐2 promotes breast cancer metastasis. J Pathol 239, 274–285. 27038000 10.1002/path.4724

[53] Hu C, Yang K, Li M, Huang W, Zhang F & Wang H (2018) Lipocalin 2: a potential therapeutic target for breast cancer metastasis. Onco Targets Ther 11, 8099–8106. 30519052 10.2147/OTT.S181223 PMC6239117

[54] Chung IH, Wu TI, Liao CJ, Hu JY, Lin YH, Tai PJ, Lai CH & Lin KH (2016) Overexpression of lipocalin 2 in human cervical cancer enhances tumor invasion. Oncotarget 7, 11113–11126. 26840566 10.18632/oncotarget.7096 PMC4905461

[55] Shao S, Cao T, Jin L, Li B, Fang H, Zhang J, Zhang Y, Hu J & Wang G (2016) Increased lipocalin‐2 contributes to the pathogenesis of psoriasis by modulating neutrophil chemotaxis and cytokine secretion. J Invest Dermatol 136, 1418–1428. 26979478 10.1016/j.jid.2016.03.002

[56] Wang G, Weng YC, Chiang IC, Huang YT, Liao YC, Chen YC, Kao CY, Liu YL, Lee TH & Chou WH (2020) Neutralization of lipocalin‐2 diminishes stroke‐reperfusion injury. Int J Mol Sci 21, 6253. 10.3390/ijms21176253 PMC7503651 32872405

