# Supplementary figures and images for: Lipocalin 2 promotes inflammatory breast cancer tumorigenesis and skin invasion
Source: Mol Oncol. 2021 Aug 27;15(10):2752–65. doi: 10.1002/1878-0261.13074 (PMC8486564; doi:10.1002/1878-0261.13074)

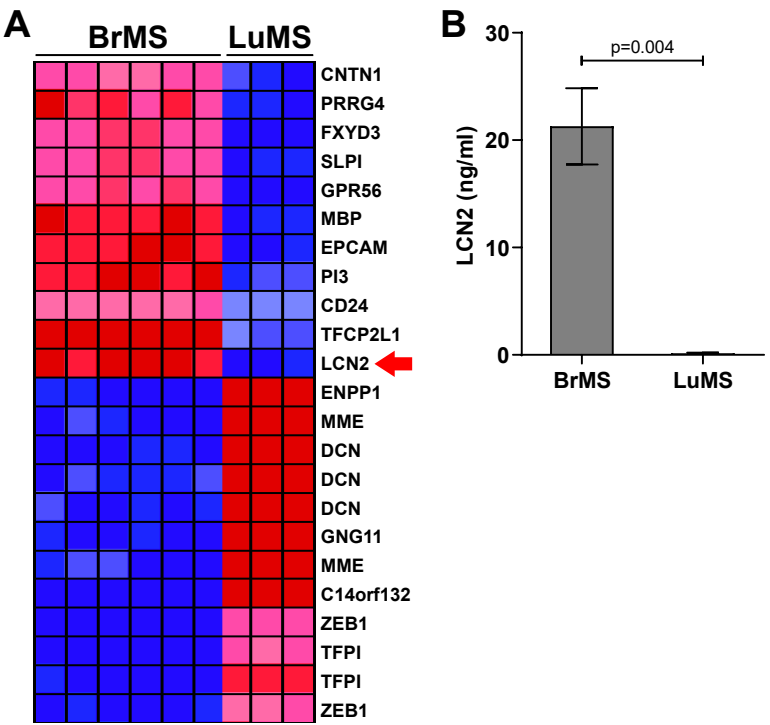

Supplement: Supplementary file 1 — Fig. S1. LCN2 expression is higher in sublines generated from brain metastasis (BrMS) than those generated from lung metastasis (LuMS). (A) Microarray analysis of sublines generated from BrMS or LuMS of SUM149 cells showed LCN2 to be one of the top upregulated genes in BrMS (red arrow). Samples are described in Debeb 2016 [29]. (B) LCN2 is secreted in higher levels in BrMS vs LuMS. [file MOL2-15-2752-s001.pdf]
