# Supplementary material for: Lipocalin 2 promotes inflammatory breast cancer tumorigenesis and skin invasion
Source: Mol Oncol. 2021 Aug 27;15(10):2752–65. doi: 10.1002/1878-0261.13074 (PMC8486564; doi:10.1002/1878-0261.13074)
Supplement: Supplementary file 2 — Table S1. Top kinases predicted to be activated based on kinase‐substrate interactions of differentially phosphorylated proteins. [file MOL2-15-2752-s002.docx]

**Supplementary Table 1**

Top kinases predicted to be activated based on kinase-substrate interactions of differentially phosphorylated proteins.

| Rank | Protein | Overlapping Protein | FET *p*-value | *FDR* | Odd Ratio |
| --- | --- | --- | --- | --- | --- |
| 1 | CSNK1D | 11 / 359 | 2.72E-12 | 9.21E-10 | 31.58 |
| 2 | MAP3K14 | 8 / 136 | 9.75E-12 | 1.65E-9 | 62.44 |
| 3 | CHUK | 13 / 773 | 6.83E-11 | 7.72E-9 | 17.09 |
| 4 | PDPK1 | 13 / 819 | 1.36E-10 | 1.15E-8 | 16.11 |
| 5 | IKBKB | 9 / 343 | 7.38E-10 | 5.00E-8 | 26.92 |
| 6 | RPS6KA4 | 8 / 256 | 1.37E-9 | 7.75E-8 | 32.49 |
| 7 | FGFR4 | 10 / 511 | 1.62E-9 | 7.85E-8 | 19.94 |
| 8 | STK3 | 7 / 180 | 3.05E-9 | 1.29E-7 | 40.42 |
| 9 | SRPK2 | 9 / 414 | 3.72E-9 | 1.40E-7 | 22.2 |
| 10 | DYRK1B | 9 / 437 | 5.9E-9 | 2.00E-7 | 21.01 |
