# Supplementary material for: Lipocalin 2 promotes inflammatory breast cancer tumorigenesis and skin invasion
Source: Mol Oncol. 2021 Aug 27;15(10):2752–65. doi: 10.1002/1878-0261.13074 (PMC8486564; doi:10.1002/1878-0261.13074)
Supplement: Supplementary file 3 — Table S2. Top kinases predicted to be activated based on kinase‐substrate and protein‐protein interaction analysis of differentially phosphorylated proteins across 10 different knowledge bases. [file MOL2-15-2752-s003.docx]

**Supplementary Table 2**

Top kinases predicted to be activated based on kinase-substrate and protein-protein interaction analysis of differentially phosphorylated proteins across 10 different knowledge bases.

| Rank | Protein | Mean rank | Overlapping Proteins |
| --- | --- | --- | --- |
| 1 | RPS6KB1 | 34.55 | 18 |
| 2 | MAPK8 | 37.91 | 19 |
| 3 | PDGFRB | 39.27 | 16 |
| 4 | MAPK9 | 42.1 | 17 |
| 5 | MAPKAPK2 | 46.78 | 15 |
| 6 | MAPK1 | 53.18 | 20 |
| 7 | RAF1 | 56.64 | 17 |
| 8 | CDK1 | 59.1 | 19 |
| 9 | MTOR | 59.45 | 18 |
| 10 | PIM1 | 60.5 | 15 |
